# Association Between Migraine Complicated With Restless Legs Syndrome and Vitamin D

**DOI:** 10.3389/fneur.2021.777721

**Published:** 2021-11-15

**Authors:** Shuning Sun, Chunling Liu, Yanlu Jia, Jun Wu, Hui Li, Xiaonan Li, Yimin Zhao

**Affiliations:** Department of Neurology, The Second Affiliated Hospital of Zhengzhou University, Zhengzhou, China

**Keywords:** migraine, restless legs syndrome, prevalence, vitamin D, case-control study

## Abstract

**Background:** This study aimed to evaluate the prevalence of restless legs syndrome (RLS) in patients with migraine and explore its association with vitamin D deficiency, aiming to provide biological support for the comorbidity of migraine with RLS, and shed new lights into clinical diagnosis and treatment.

**Methods:** A case-control study was performed on 175 migraine patients and 151 non-headache controls. The information of all subjects concerning headache severity [visual analog scale (VAS) score], RLS, RLS severity [International Restless Legs Scale (IRLS) score], sleep quality [Pittsburgh sleep quality index (PSQI)], anxiety and depression symptoms [hospital anxiety and depression scale (HADS)], and demographic data were collected. At the same time, serum 25-(OH) D levels were also measured (concentration <20 ng/ml was defined deficiency). Afterward, the logistic regression model was adopted to explore the risk factors for RLS in patients with migraines.

**Results:** Compared with control group, migraine group had lower vitamin D levels [(21.10 ± 6.58) vs. (16.42 ± 5.6) ng/ml, *P* < 0.001], a higher rate of vitamin D deficiency (45.03 vs. 72%, *P* <0001), higher prevalence of RLS (6.62 vs. 22.29%, *P* < 0.001). Compared with the pure RLS group, RLS with the migraine group had lower vitamin D levels and higher IRLS score (*P* < 0.05). Compared with pure migraine group, migraine with RLS group had lower vitamin D levels [(17.36 ± 5.56) vs. (13.15 ± 4.42) ng/ml, *P* < 0.001], higher incidence of vitamin D deficiency (66.18 vs. 92.31%, *P* = 0.001), higher frequency of headache attacks (*P* = 0.004). Thereafter, the multivariate logistic regression model was employed to adjust confounding factors such as age, gender, season, frequency of headache attacks, PSQI score, and HADS scores. According to the results vitamin D deficiency in patients with migraines was an independent risk factor for RLS (OR = 5.03, 95%CI: 1.2–21.16, *P* = 0.027).

**Conclusions:** The prevalence of RLS in migraine patients was significantly higher than that in the non-headache population. Besides, vitamin D levels decreased, while the incidence of vitamin D deficiency increased in the migraine patients complicated with RLS. Finally, the occurrence of RLS in migraine patients was significantly related to vitamin D deficiency.

## Introduction

Migraine refers to a kind of chronic disabling neurological disease, which has affected one-tenth of the global population (1). It is characterized by recurrent unilateral throbbing pain aggravated by physical activity and is accompanied by symptoms such as photophobia, phonophobia, nausea, and vomiting (2). Studies have found that migraine may be associated with a variety of concomitant diseases, including cardiovascular disease (CVD), asthma, depression, stroke, epilepsy, and other painful diseases (3). In addition, sleep disturbance is also common comorbidity of migraines. Restless legs syndrome (RLS) is specifically related to migraines (4). RLS is a common sensory-motor disorder of the nervous system, which is characterized by a strong desire to move the legs to relieve discomfort. It usually occurs at rest and often worsens at night. The symptoms are relieved after moving the limbs (4). Tiseo et al. pointed out that the prevalence of RLS in migraine patients was about 13.7–25%, and that of migraine in RLS was about 12.6–53.2%, and they were significantly higher than those in healthy controls (5).

The comorbid mechanism of migraine and RLS remains unclear at present. Currently, more and more studies show that the two share common pathophysiological mechanisms in dopaminergic imbalance, iron metabolism disorders, genetic mutations, serotonergic disorders, sleep disorders, anxiety and depression, and structural imaging (6–8). Recent studies have found that the serum vitamin D levels in migraine patients are lower than those in the normal population (9–12), and vitamin D supplementation can reduce the frequency of migraine attacks, alleviate the headache severity and improve the disability symptoms (13, 14). In addition, some case-control studies discover that the incidence of RLS in the vitamin D deficiency group is higher than that in the normal vitamin D group, and the vitamin D level is negatively correlated with the severity of RLS (15–17). Therefore, it remains to be further explored whether the comorbid mechanism of migraine and RLS is related to serum vitamin D deficiency. In this study, the vitamin D levels were observed in migraine patients complicated with RLS and in patients with pure migraine, so as to explore the association between the occurrence of RLS and vitamin D deficiency in migraine patients. Findings in this study could provide biological support for the comorbid mechanism of migraine and RLS, thereby providing new ideas for clinical diagnosis and treatment.

## Methods

### Study Design and Population

**Figure 1:**
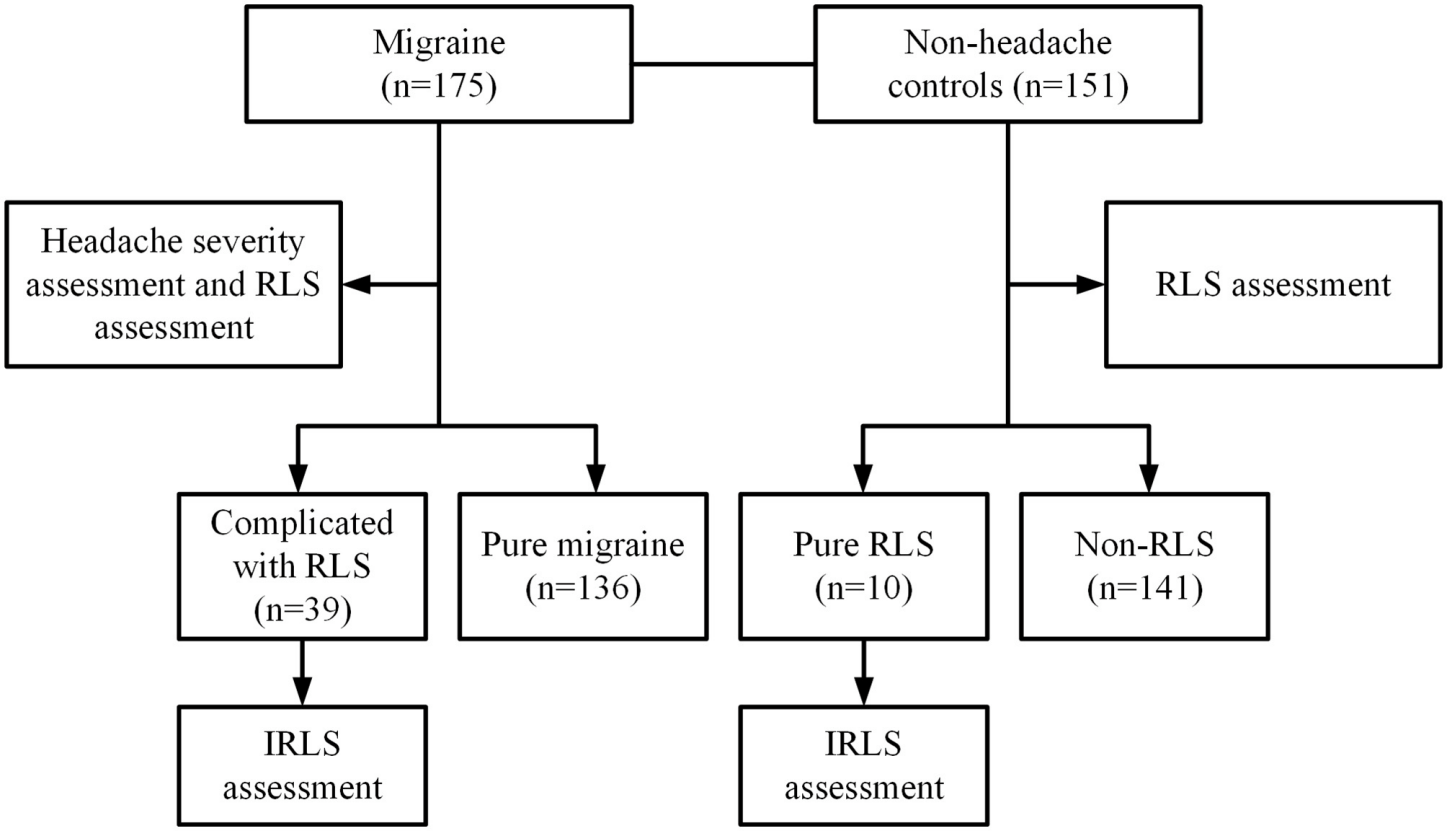
Flow chart of the study. This study collected 175 migraine patients who met the diagnostic criteria of the International Classification of Headache Disorders (ICHD-III b) at the Department of Neurology of the Second Affiliated Hospital of Zhengzhou University from January 2019 to June 2021 and 151 age-matched non-headache controls who underwent physical examinations at the Physical Examination Center during the same period. All subjects were evaluated for RLS. And migraine patients were also evaluated for headache severity. Finally, there were 39 (22.29%) migraine patients complicated with RLS and 136 (77.71%) pure migraine among the migraine patients, and 10 (6.62%) pure RLS and 141 (93.38%) non-RLS subjects among non-headache controls.

This case-control study collected migraine patients diagnosed at the Department of Neurology of our hospital from January 2019 to June 2021. The patient inclusion criteria were as follows; the migraine group should include patients aged 18–60 years who met the diagnostic criteria for migraine (2013) released by the International Headache Association (ICHD-III) (18). Patients conforming to any one of the following criteria were excluded: patients with diseases that affected vitamin D levels or metabolisms, such as infectious diseases, liver and kidney diseases, gastrointestinal diseases, cancer, sarcoidosis, osteoporosis, osteomalacia, and thyroid diseases; patients with a history of treatment with corticosteroids, diuretics, statins, estrogen, bisphosphonates, calcitonin, calcium or vitamin D; patients with secondary causes of RLS like Parkinson's disease (PD), peripheral neuropathy, liver and kidney failure, pregnancy, abnormal vitamin B12, and ferritin levels. Additionally, the age-matched controls who underwent physical examinations at the Physical Examination Center of our hospital during the same period were recruited. The control subjects never had primary headache disorders defined by ICHD-III, at the same time meeting the above exclusion criteria. According to the above criteria, 175 migraine patients (including 47 men and 128 women; age 40.2 ± 8.63 years) and 151 non-headache controls (including 46 men and 105 women; age 41.7 ± 8.48 years) were finally included. The flowchart of this study is shown in Figure 1. This study was approved by the Ethics Committee of the Second Affiliated Hospital of Zhengzhou University. Written informed consent was obtained from each subject.

### Data Collection

#### Clinical Assessment

The general clinical data like demographic indicators were collected, and the visit seasons of subjects were recorded and divided into spring (March–May), summer (June–August), autumn (September–November), and winter (December–February). In addition, the diagnosis of migraine, migraine characteristics, the diagnosis of RLS, RLS severity, and some questionnaires were evaluated by two experienced neurologists specialized in headache and sleep. Because they had difficulty in filling out the questionnaires when the headache attacked, we evaluated them on the second day after the headache was relieved. For the assessment of migraine severity, visual analog scale (VAS) is used to assess the degree of pain (0–10 means the severity of pain, where “0” means no such symptoms, while “10” indicates the most severe symptoms). For the assessment of RLS, the professional physician doctor evaluated each subject in line with the RLS diagnostic criteria developed by the International Restless Legs Syndrome Study Group (IRLSSG) (19). For patients who met the diagnostic criteria, we used the International Restless Legs Scale (IRLS) to assess RLS severity within the past week (20). The total score of the scale is 40 points, with 1–10 being mild, 11–20 being moderate, 21–30 being severe, and 31–40 being very severe (21). For the assessment of sleep quality, the Pittsburgh sleep quality index (PSQI) has been widely used to measure the quality of sleep in the past month, which includes sleep quality, sleep time, sleep efficiency, sleep latency, sleep disorders, hypnosis, and daytime dysfunction. The subjects filled in the form according to their sleep status in the past month. The scores range from 0 to 21, and a total score of >5 indicates poor sleep quality. For the assessment of anxiety and depression, the hospital anxiety and depression scale (HADS) consists of two subscales for anxiety and depression, with a starting point of 8 points, wherein a score of ≥8 points is considered as suspicious or symptomatic.

#### Determination of Serum Vitamin D Levels

Vitamin D level is usually assessed by measuring the serum 25-(OH) D level. In this study, the nurse from the Neurology Department of our hospital collected fasting venous blood from the elbow vein of each subject from 6:00 to 8:00 a.m. on the second day after the headache was relieved. Furthermore, 3–4 ml blood samples were collected into the ordinary glass tubes, centrifuged within 1 h, and stored at −20°C, and then the electrochemiluminescence method was employed to determine the serum 25-(OH) D levels. Vitamin D deficiency was defined as the 25-(OH) D concentration <20 ng/ml, and vitamin D level ≥20 ng/ml was considered normal.

### Statistical Analysis

All data were explored using IBM SPSS Statistics V21.0 (IBM, Armonk, New York). For continuous data, an independent sample *t-test* was used to analyze normally distributed variables and data with equal variances; otherwise, the Mann-Whitney *U* test was adopted. The Chi-square test was utilized to analyze the categorical variables. Normally distributed data were expressed as *M* ± *SD*, while the abnormally distributed data as median (quartile) [M (Q25, Q75)]. Regarding normally distributed data, we used Pearson correlation analysis to analyze the correlation between vitamin D levels and headache severity and/or RLS severity. Otherwise, we employed Spearman correlation analysis for non-normally distributed data. Age, season, anxiety, depression, sleep quality, and disease course were incorporated as covariates in the analysis due to their potential influences on vitamin D level, migraine severity, and RLS severity. Moreover, the logistic regression model was used to analyze the risk factors for RLS in migraines. The multivariate analysis was adjusted for age, gender, body mass index (BMI), education level, smoking, drinking, coffee consumption, season, VAS score, frequency of headache attacks, disease course, PSQI score, and HADS score. In terms of seasons, the vitamin D level showed periodic seasonal changes, which increased from May to September and decreased from November to March (22). Therefore, we divided the seasons into spring/winter and summer/autumn. Odds ratio (OR) with 95% CI were reported. The statistical significance level was set at *p* < 0.05. All reported *p*-values were two-sided.

## Results

### Clinical Data and Vitamin D Level of Migraine Group and Control Group

Differences in age, gender, BMI, education level, smoking, alcohol consumption, and season were not significant between the migraine group and the control group (all *p* > 0.05). The coffee consumption, PSQI score, HADS-anxiety score, and HADS-depression score in the migraine group were higher than those in the control group (all *p* < 0.05). Additionally, the vitamin D deficiency rate (72 vs. 45.03%, *p* < 0.001) and the prevalence of RLS (22.29 vs. 6.62%, *p* < 0.001) in the migraine group were higher than those in the control group. The serum vitamin D levels of the migraine group were lower than those of the control group (*p* < 0.001; Table 1).

**Table 1:** Clinical data and vitamin D levels between migraine group and control group.

	**Migraine (*n* = 175)**	**Control (*n* = 151)**	***t*/*Z*/χ^**2**^-value**	***P*-value**
Age (years)	40.20 ± 8.63	41.70 ± 8.48	−1.586^a^	0.114
Female, *n* (%)	128 (73.14)	105 (69.54)	0.517^b^	0.472
BMI (kg/m^2^)	23.20 ± 2.19	22.77 ± 2.39	1.696^a^	0.091
Education level (years)	14.00 (10.00, 17.00)	12.00 (9.00, 16.00)	−1.560^c^	0.119
Smoking status, *n* (%)	33 (18.86)	23 (15.23)	0.749^b^	0.387
Alcohol consumption, *n* (%)	38 (21.71)	26 (17.22)	1.038^b^	0.308
Coffee consumption, *n* (%)	51 (29.14)	28 (18.54)	4.960^b^	0.026
**Season**			2.046^b^	0.563
Spring, *n* (%)	47 (26.86)	36(23.84)		
Summer, *n* (%)	34 (19.43)	25(16.56)		
Autumn, *n* (%)	43 (24.57)	35(23.18)		
Winter, *n* (%)	51 (29.14)	55(36.42)		
**Migraine**				
Age of onset (years)	28.06 ± 7.28	–		
Course of disease (years)	12.00 (6.00, 17.00)	–		
VAS score	7.00 (6.00, 8.00)	–		
Monthly days with headache	9.06 ± 3.79	–		
PSQI total score	7.00 (4.00, 9.00)	6.00 (4.00, 8.00)	−3.214^c^	0.001
HADS-anxiety	8.44 ± 3.44	6.81 ± 2.91	−4.262^c^	<0.001
HADS-depression	7.62 ± 3.17	5.85 ± 2.89	5.226^a^	<0.001
25-(OH) D (ng/mL)	16.42 ± 5.60	21.10 ± 6.58	−6.148^c^	<0.001
Vitamin D deficiency, *n* (%)	126 (72.00)	68 (45.03)	24.463^b^	<0.001
RLS, *n* (%)	39 (22.29)	10 (6.62)	15.571^b^	<0.001

a *t-value obtained by t-test;*

b *χ^2^-value obtained by Chi-square test;*

c *Z-value obtained by Mann-Whitney U-test*.

*BMI, body mass index; VAS, visual analog scale; RLS: restless legs syndrome; PSQI, Pittsburgh sleep quality index; HADS, hospital anxiety and depression scale*.

### The IRLS Score and Vitamin D Levels Between Migraine and RLS Comorbidity Group and Pure RLS Group

There were 39/175 confirmed RLS patients in the migraine group, and 10/151 confirmed RLS patients in non-headache controls. Differences in age of onset of RLS and course of RLS were not significant between the migraine patients complicated with RLS and the RLS patients among controls (pure RLS patients) (both *p* > 0.05). The IRLS score in the migraine patients complicated with RLS was higher than those in the pure RLS patients (*p* = 0.007). The serum vitamin D levels of migraine and RLS comorbidity groups were lower than those of the pure RLS group (*p* = 0.002; Table 2).

**Table 2:** The IRLS score and vitamin D levels between migraine and RLS comorbidity group and pure RLS group.

	**Migraine and RLS comorbidity (*n* = 39)**	**Pure RLS (*n* = 10)**	***t*/*Z*-value**	***P*-value**
Age of onset (years)	31.56 ± 7.44	33.90 ± 7.98	−0.874^a^	0.387
Course of disease (years)	10.41 ± 6.89	7.10 ± 3.60	−1.318^b^	0.188
IRLS score	16.64 ± 5.92	10.90 ± 5.24	2.795^a^	0.007
25-(OH)D (ng/mL)	13.15 ± 4.42	18.58 ± 5.49	−3.297^a^	0.002

a *t-value obtained by t-test;*

b *Z-value obtained by Mann-Whitney U-test*.

*RLS: restless legs syndrome; IRLS, International restless legs scale*.

### Clinical Data and Vitamin D Levels Between Pure Migraine Group and Migraine and RLS Comorbidity Group

The monthly days with headache, PSQI score, HADS-anxiety, HADS-depression score, and vitamin D deficiency rate of the migraine and RLS comorbidity group were higher than those of the pure migraine group (all *p* < 0.05). In addition, the vitamin D levels of the migraine and RLS comorbidity group were significantly lower than those of the pure migraine group (*p* < 0.001; Table 3).

**Table 3:** Clinical data and vitamin D levels between migraine and RLS comorbidity group and pure migraine group.

	**Migraine and RLS comorbidity (*n* = 39)**	**Pure migraine (*n* = 136)**	***t*/*Z*/χ^**2**^-value**	***P*-value**
Age (years)	41.97 ± 7.89	39.69 ± 8.80	1.461^a^	0.146
Female, *n* (%)	29 (74.36)	99 (72.79)	0.038^b^	0.846
BMI (kg/m^2^)	22.86 ± 2.33	23.30 ± 2.15	−1.095^a^	0.275
Education level (years)	15.00 (12.00, 17.00)	13.00 (9.00, 17.00)	−1.294^c^	0.196
Smoking status, *n* (%)	7 (17.95)	26 (19.12)	0.027^b^	0.869
Alcohol consumption, *n* (%)	7 (17.95)	31 (22.79)	0.419^b^	0.518
Coffee consumption, *n* (%)	12 (30.77)	39 (28.68)	0.064^b^	0.800
**Season**			2.292^b^	0.514
Spring, *n* (%)	9 (23.08)	38 (27.94)		
Summer, *n* (%)	9 (23.08)	25 (18.38)		
Autumn, *n* (%)	7 (17.95)	36 (26.47)		
Winter, *n* (%)	14 (35.90)	37 (27.21)		
**Migraine**				
Age of onset (years)	29.15 ± 8.04	27.74 ± 7.05	1.068^a^	0.287
Course of disease (years)	12.00 (7.00, 17.00)	11.50 (6.00, 17.00)	−0.829^c^	0.407
VAS score	7.41 ± 1.45	7.06 ± 1.64	1.212^a^	0.227
Monthly days with headache	10.59 ± 4.04	8.62 ± 3.61	2.927^a^	0.004
PSQI total score	8.31 ± 3.66	6.38 ± 3.02	3.355^a^	0.001
HADS-anxiety	9.54 ± 2.87	8.13 ± 3.53	2.289^a^	0.023
HADS-depression	9.33 ± 3.17	7.13 ± 3.01	3.979^a^	<0.001
25-(OH) D (ng/mL)	13.15 ± 4.42	17.36 ± 5.56	−4.345^a^	<0.001
Vitamin D deficiency, *n* (%)	36 (92.31)	90 (66.18)	10.266^b^	0.001

a *t-value obtained by t-test;*

b *χ^2^-value obtained by Chi-square test;*

c *Z-value obtained by Mann-Whitney U-test*.

*BMI, body mass index; VAS, visual analog scale; RLS: restless legs syndrome; PSQI, Pittsburgh sleep quality index; HADS, hospital anxiety and depression scale*.

### Correlation Between Severity of Disease and Vitamin D Levels

The monthly days with headache and IRLS score were negatively correlated with vitamin D levels, after adjusting for age, season, HADS score, PSQI score, and course of disease (Table 4).

**Table 4:** Correlation between severity of disease and vitamin D levels.

	**Vitamin D levels (unadjusted)**		**Vitamin D levels (adjusted)**	
	***r***	***P***	***r***	***p***
VAS score	−0.141	0.062	−0.139	0.072
Monthly days with headache	−0.218	0.004	−0.239	0.002
IRLS score	−0.440	0.002	−0.368	0.015

*VAS, visual analog scale; IRLS, International Restless Legs Scale; Adjusted, adjusting for age, season, HADS score, PSQI score, and course of the disease*.

### Risk Factors for Migraine and RLS Comorbidity

As revealed by univariate logistic regression analysis, the monthly days with headache, high PSQI score, high HADS score and vitamin D deficiency were significantly associated with the occurrence of RLS in migraine patients. Moreover, according to multivariate regression analysis adjusted for age, gender, BMI, season, course of migraine, VAS score, monthly days with headache, PSQI score, HADS score, and other confounding factors, vitamin D deficiency was still related to RLS (OR = 5.03, 95%CI: 1.20–21.16, *p* = 0.027; Table 5).

**Table 5:** Risk factors of RLS in migraine patients with logistic regression analysis.

	**Univariate analysis**		**Multivariate analysis**	
	**OR (95% CI)**	***P*-value**	**OR (95% CI)**	***P-*value**
Age (years)	1.03 (0.99–1.08)	0.147	1.06 (0.99–1.13)	0.094
Female	1.08 (0.48–2.44)	0.846	0.53 (0.08–3.44)	0.506
BMI (kg/m^2^)	0.91 (0.78–1.08)	0.274	0.93 (0.75–1.15)	0.495
Education level (years)	1.06 (0.98–1.16)	0.166	1.06 (0.95–1.17)	0.285
Smoking status	0.93 (0.37–2.33)	0.869	0.78(0.13–4.54)	0.778
Alcohol consumption	0.74 (0.30–1.84)	0.519	0.49 (0.11–2.30)	0.369
Coffee consumption	1.11 (0.51–2.40)	0.800	1.59 (0.58–4.31)	0.365
Spring/Winter	1.17 (0.57–2.41)	0.671	0.88 (0.35–2.18)	0.780
Course of migraine (years)	1.02 (0.97–1.06)	0.527	0.98 (0.91–1.05)	0.556
VAS score	1.15 (0.92–1.44)	0.227	0.99 (0.73–1.33)	0.940
Monthly days with headache	1.15 (1.04–1.27)	0.005	1.17 (1.02–1.33)	0.022
PSQI total score	1.21 (1.08–1.36)	0.002	1.20 (1.06–1.37)	0.005
HADS-anxiety	1.13 (1.02–1.26)	0.025	1.12 (0.98–1.27)	0.093
HADS-depression	1.27 (1.12–1.44)	<0.001	1.28 (1.10–1.49)	0.001
Vitamin D deficiency	6.13 (1.79–20.99)	0.004	5.03 (1.20–21.16)	0.027

*RLS, restless legs syndrome; BMI, body mass index; VAS, visual analog scale; PSQI, Pittsburgh sleep quality index; HADS, hospital anxiety and depression scale; Spring/winter, spring, and winter are regarded as one category, and the rest of autumn and summer is regarded as another category*.

## Discussion

This was a single-center study with a small sample size. Although larger multicenter studies are needed, our study strongly suggests that vitamin D deficiency is an independent risk factor for RLS in migraine sufferers. In recent years, widespread attention has been paid to the comorbid association between migraine and RLS. This study found that the prevalence of RLS in migraine patients (22.29%) was significantly higher than that in non-headache controls (6.62%), which was consistent with other research findings (3, 6, 21, 23, 24). In addition, it has also been found that migraines and RLS affect each other. Compared with patients with pure RLS, patients with migraines are associated with more severe RLS symptoms (21), which is consistent with our result. Suzuki et al. found that migraine patients with RLS have higher scores on the Migraine Disability Assessment Questionnaire (MIDAS) compared with those of pure migraine patients (23). However, we found that migraine complicated with the RLS group had more headache attacks per month than those in the pure migraine group, but there was no difference in the severity of headache. At the same time, vitamin D levels were lower in the migraine complicated with the RLS group, and we found that vitamin D levels were negatively correlated with the frequency of migraine attacks, but it did not correlate with the VAS score. Therefore, we speculate that the higher frequency of headaches in the migraine complicated with the RLS group is related to lower vitamin D levels. However, there is conflicting result regarding the relationship between vitamin D levels and the frequency of migraine attacks and headache severity (9–11), and a larger sample should be studied. Similarly, more severe RLS symptoms in migraine patients may also be related to vitamin D, because vitamin D levels are also negatively correlated with IRLS scores. Therefore, it is of great significance to understand the comorbid mechanism of migraine and RLS, aiming to reduce the occurrence of this comorbidity and improve the quality of life of patients.

At present, there have been many reports on the respective relationship of vitamin D level with migraine (10–12) and RLS (15–17, 25), but the comorbid mechanism of migraine and RLS remains unclear. Therefore, we speculate that vitamin D level is related to the mechanism of this comorbidity and conduct related research. According to reports, the rate of vitamin D deficiency in headache patients is 45–100% (26). Prakash found that as the latitude increased, the prevalence of migraine also showed an increasing trend, and the overall frequency of migraine attacks was the highest in winter but the lowest in summer (22). This headache pattern seems to match with the seasonal changes in vitamin D level, which rises from May to September, and are lower from November to March (22). In this study, the number of migraine patients who visited the clinic was the highest in winter and the lowest in summer, which was consistent with the distribution of the appeal season. We found that the vitamin D levels in the migraine group were reduced, and the vitamin D deficiency rate (72%) was significantly higher than that of the control group (45.03%), consistent with previous studies (9–12). In addition, this study suggested that the vitamin D level, vitamin D deficiency rate, frequency of headache attacks, sleep quality, anxiety, and depression levels in the migraine complicated with RLS group were more serious than those in the pure migraine group. Therefore, it was speculated that serum vitamin D level was related to migraines complicated with RLS.

To exclude the impacts of frequency of headache attacks, season, sleep quality, anxiety, and depression on the occurrence of RLS, the logistics regression model was utilized to analyze the related factors for RLS. Univariate analysis revealed that the monthly days with headache, high PSQI score, high HADS score, and vitamin D deficiency were the risk factors for RLS in patients with migraines. Firstly, in terms of sleep quality, anxiety, and depression, a large number of previous studies have reported that migraine patients complicated with RLS have worse sleep quality and more significant anxiety and depression symptoms than those with migraine alone (3, 7, 21, 23, 24). Besides, these clinical factors are related to the occurrence of RLS (3, 7, 21), consistent with our findings. This may be related to the severe nighttime symptoms of RLS patients, which affect the sleep quality and further aggravate the anxiety and depression symptoms. Secondly, in terms of monthly days with headache, our results were consistent with Lin et al. who found that higher migraine frequency correlated with the higher prevalence of RLS (3). Frequent headache attacks may exacerbate anxiety, depression, poor sleep mutually, and promote the occurrence of RLS. Finally, after adjusting for the confounding factors, we found that vitamin D deficiency was still an independent risk factor for RLS in patients with migraines. Therefore, we speculated that vitamin D deficiency might play an important role in the comorbidity of migraine and RLS.

At present, numerous related studies have reported the role of vitamin D in migraine and RLS (11, 16, 17, 26, 27). This study aimed to explore the role of vitamin D in the comorbidity of migraine and RLS, and the following aspects are speculated. (1) In terms of dopaminergic disorder, central dopaminergic dysfunction is one of the widely recognized pathogenic mechanisms of RLS. In addition, dopaminergic nucleus dysfunction may cause or aggravate migraine by promoting the discharge of the trigeminal nerve complex (23), and dopamine receptor agonists are effective in reducing the frequency of migraine attacks (2). Studies have found that vitamin D can increase the levels of dopamine or its metabolites in the brain, and up-regulate the levels of glutathione and neurotrophin synthetase by inhibiting nitric oxide (NO) synthesis, which protects dopaminergic neurons (15, 28, 29). Therefore, the reduction in vitamin D levels leads to dopaminergic disorders, triggers migraine attacks and RLS, increases the NO level, and aggravates the migraine symptoms. (2) With regard to serotonergic disorder, it is known that serotonergic disorder is involved in the pathogenesis of migraine (27), which also causes RLS directly or indirectly through interacting with dopamine (23). Valente et al. considered that serotonergic overload was associated with the occurrence of RLS in migraine patients (6). Vitamin D can affect the synthesis of serotonin via tyrosine hydroxylase and subsequently, participate in the occurrence of the comorbidity of migraine and RLS. (3) From the perspective of central sensitization, on the one hand, central sensitization is involved in the pathogenesis of migraines. The dural inflammation continuously activates the trigeminal neurovascular system and leads to peripheral sensitization, which then extends to the trigeminal nuclei and even the thalamic neurons to cause central sensitization (30). On the other hand, it is found that patients with RLS also have neurogenic hyperalgesia mediated by central sensitization, which is characterized by the increased excitability of nociceptive spinal cord neurons. Moreover, it may involve the basal ganglia and/or descending dopaminergic pathways (31). Vitamin D exerts its anti-inflammatory and analgesic effects by reducing the release of pro-inflammatory cytokines and inhibiting T-cell responses (10, 26, 27). Apart from that, vitamin D participates in calcium signal transduction in nerve cells, promotes the production of reactive oxygen species (ROS) and neurotrophic factors, and inhibits both central and peripheral nerve sensitization (9).

In summary, the comorbidity of migraine and RLS may be related to vitamin D deficiency. This study sheds new light on the pathogenesis of migraines complicated with RLS, which is of great significance for the clinical guidance of these patients. For migraine patients, it is necessary to measure the serum 25-(OH) D levels, especially for patients with concomitant RLS. Obviously, timely supplementation in the case of vitamin D deficiency may reduce the occurrence of RLS comorbidities or alleviate the severity of RLS. Of course, certain limitations should be noted in this study. Firstly, this study was an observational study. In the future, therapeutic research can be conducted to further explore the effect of vitamin D on RLS. Secondly, when assessing the severity of migraines, we only used the VAS questionnaire. In the future, we can assess MIDAS, HIT-6 as well as other questionnaires to further improve our research results. Thirdly, although the most important confounding factors were controlled when evaluating the RLS-related factors, it was still impossible to rule out the influence of all potential confounding factors on the experimental results. Fourthly, it would be important to evaluate the changes in the VAS score, frequency of headache attacks, and IRLS score in the same patient during the year (due to the seasonability of vitamin D level), but we only evaluated the seasonal distribution of the visits of patients. Finally, the sample size of the study was small, and a multicenter study with a large sample size should be conducted in the future.

## Data Availability Statement

The raw data supporting the conclusions of this article will be made available by the authors, without undue reservation.

## Ethics Statement

The studies involving human participants were reviewed and approved by Ethics Committee of the Second Affiliated Hospital of Zhengzhou University. The patients/participants provided their written informed consent to participate in this study.

## Author Contributions

CL and SS designed the study. Material preparation, data collection, and analysis were performed by SS, HL, YJ, JW, XL, and YZ. SS drafted the initial manuscript. All authors commented on previous versions of the manuscript, read, and approved the final manuscript.

## Funding

This study was supported by Henan Province College and University Innovation Talent Project (182102310586).

## Conflict of Interest

The authors declare that the research was conducted in the absence of any commercial or financial relationships that could be construed as a potential conflict of interest.

## Publisher's Note

All claims expressed in this article are solely those of the authors and do not necessarily represent those of their affiliated organizations, or those of the publisher, the editors and the reviewers. Any product that may be evaluated in this article, or claim that may be made by its manufacturer, is not guaranteed or endorsed by the publisher.
